# Acetylation of Lysine 243 Inhibits the *oriC* Binding Ability of DnaA in *Escherichia coli*

**DOI:** 10.3389/fmicb.2017.00699

**Published:** 2017-04-20

**Authors:** Shuxian Li, Qiufen Zhang, Zhihong Xu, Yu-Feng Yao

**Affiliations:** ^1^Laboratory of Bacterial Pathogenesis, Institutes of Medical Sciences, Shanghai Jiao Tong University School of MedicineShanghai, China; ^2^Department of Laboratory Medicine, Shanghai East Hospital, Tongji University School of MedicineShanghai, China

**Keywords:** lysine acetylation, replication initiation, initiator DnaA, acetyl-phosphate, *Escherichia coli*

## Abstract

DNA replication initiation is a central event in the cell cycle, and it is strictly controlled by multiple regulatory mechanisms. Our previous work showed that acetylation of residue lysine (K) 178 prevents DnaA from binding to ATP, which leads to the inhibition of DNA replication initiation. Here, we show that another residue, K243, is critical for DnaA full activity *in vivo*. K243 can be acetylated, and its acetylation level varies with cell growth. A homogeneous, recombinant DnaA that contains N^𝜀^-acetyllysine at K243 (K243Ac) retained its ATP/ADP binding ability, but showed decreased binding activity to the *oriC* region. A DNase I footprinting assay showed that DnaA K243Ac failed to recognize DnaA boxes I3, C1, and C3, and, thus, it formed an incomplete initiation complex with *oriC*. Finally, we found that acetyl phosphate and the deacetylase CobB can regulate the acetylation level of K243 *in vivo*. These findings suggest that DnaA K243 acetylation disturbs its binding to low-affinity DnaA boxes, and they provide new insights into the regulatory mechanisms of DNA replication initiation.

## Introduction

Chromosome replication initiation is a central event in the bacterial cell cycle, and the initiator DnaA plays essential roles in this process (Kornberg and Baker, 1992; Messer, 2002; Mott and Berger, 2007). In *Escherichia coli*, DnaA forms complex with ATP/ADP, and it binds to the replication origin (*oriC*). This interaction leads to a local unwinding in an adjacent AT-rich region, which is assisted by the DNA-structuring proteins HU or integration host factor (IHF) (Hwang and Kornberg, 1992). The unwound region provides an entry site for the DnaB-DnaC helicase complex in a manner mediated by DnaC and an *oriC*-bound DnaA interaction. Then, DnaB migrates along the DNA to expand the region of single-stranded DNA (Kornberg and Baker, 1992), which leads to the assembly of replication machineries, including the DnaG primase and the DNA polymerase III holoenzyme (O’Donnell, 2006).

Binding of DnaA to the replication origin is the first step of nascent DNA synthesis in all systems (Baker and Bell, 1998). *oriC* of *E. coli* contains, within 260 bp, five copies of 9-mer DnaA binding sites (R boxes, R1–R5) with the consensus sequence 5′–TT(A/T)TNCACA–3′ (Schaper and Messer, 1995). Although differing slightly in their nucleotide sequences, these DnaA boxes have different binding affinities for DnaA (Sekimizu et al., 1987; Schaper and Messer, 1995). Speck and Messer (2001) found that ATP-DnaA but not ADP-DnaA adopts a new binding specificity for the consensus sequence 5′–AG(A)(T)(C)(T)–3′. These 6-mer ATP-DnaA boxes located in the AT-rich DNA-unwinding element (DUE) are bound cooperatively by ATP-DnaA, which promotes unwinding of the DUE region (Speck and Messer, 2001). ATP-DnaA also binds to another class of sequence flanking the center of *oriC*, termed I sites, which have the consensus sequence 5′–(A/T)G(G/C)(A/T)N(G/C)G(A/T)(A/T) (T/C)A–3′ (Grimwade et al., 2000; McGarry et al., 2004). Subtly different from the R box, DnaA interactions with these sites are enhanced in the presence of IHF. Although with low affinity for ATP-DnaA binding, I sites are required for DNA strand opening (Grimwade et al., 2000; McGarry et al., 2004). Furthermore, Kawakami et al. (2005) found τ sites (τ1 and τ2) sharing sequence homology with the I2 and I3 sites, while Rozgaja et al. (2011) reported C sites (C1, C2, and C3). All the low-affinity sites together provide nucleotide-based instructions for the ordered assembly of DnaA oligomers (Rozgaja et al., 2011). DnaA interaction with *oriC* is cooperative, and it occurs in a specific order, with high-affinity sites (R4, R1, and R2) filled throughout most of the cell cycle, while low-affinity sites are only filled immediately before the onset of DNA synthesis (Ryan et al., 2004). DnaA binding to low-affinity sites is more important in triggering initiation, but the key DnaA residues that are responsible for recognizing low-affinity sites are less understood.

As the genetic information carrier, the replication of DNA must be limited to once per cell cycle, and it must occur at the correct time. Multiple mechanisms regulate this process at the initiation stage (Mott and Berger, 2007; Katayama et al., 2010). Our previous work showed that acetylation of DnaA at the conserved lysine (K) residue 178 in the Walker A motif inhibited its ATP/ADP binding ability and its subsequent *oriC* binding activity (Zhang et al., 2016). In this study, we found that acetylation of K243 inhibits the *oriC* binding activity of DnaA, but it does not affect the ATP/ADP binding affinity of DnaA or the ability of DnaA to bind the *dnaA* promoter region and the DnaA-reactivating sequence 1 (DARS1). Our data suggest that acetylation negatively regulates DNA replication initiation by disturbing the binding of DnaA to low-affinity boxes, which increases our understanding of this precise replication initiation process.

## Materials and Methods

### Bacterial Strains, Plasmids, Primers, and Media

All the bacterial strains, plasmids, and primers used in this study are listed in Supplementary Tables S1, S2.

### Protein Purification Procedure

The site-specific acetylated DnaA (K243Ac) was purified as previously described (Ren et al., 2016; Zhang et al., 2016) with some modifications. 1 L culture of *E. coli* strain BL21 carrying plasmid pAcKRS-3 and pCDF-PylT-*dn*aA (K243TAG) was grown at 37°C in LB medium supplemented with streptomycin (50 μg/mL) and kanamycin (50 μg/mL). At an OD_600_ = 1.6–1.8, 1 L LB with 20 mM acetyl-lysine (AcK) was added. DnaA K243Ac was induced overnight by adding IPTG at a final concentration of 1 mM.

The harvested cells were resuspended in cold buffer A (50 mM Tris-HCl, pH 7.5, 500 mM NaCl, 20 mM imidazole, 10% glycerol), cell suspension was lysed by pressure cell disrupters. The lysate was centrifuged to collect the supernatant, ammonium sulfate was added slowly at a final concentration of 0.22 g/ml, and the resulting precipitate was collected by centrifugation, dissolved with buffer A. After dialyzed overnight against the same buffer, the solution was loaded to a 1 ml Ni-NTA column (GE Healthcare) pre-washed with buffer A. After washing with buffer A, the protein was eluted with buffer B (50 mM Tris-HCl, pH 7.5, 500 mM NaCl, 500 mM imidazole, 10% glycerol), fractions containing DnaA K243Ac were desalted using a 5 ml HiTrap desalting column (GE Healthcare) pre-washed with buffer D (50 mM HEPES-KOH, pH7.5, 0.1 mM EDTA, 20% sucrose, 2 mM DTT, 0.2 M ammonium sulfate, 10 mM magnesium). The wild-type DnaA was purified in the same way, protein at purity > 90% (Supplementary Figure S1) was kept frozen at -80°C.

The purification of YfiQ, CobB and native DnaA proteins were the same as previously described (Zhang et al., 2016).

### Plasmid Complementation Assay

This assay was performed mainly as described (Kawakami et al., 2005). KA413 [*dnaA*46(Ts)] cells were transformed with 250 ng plasmid bearing the indicated *dnaA* allele and incubated on LB agar containing thymine (50 μg/ml) and spectinomycin (100 μg/mL) at 30°C or plated on LB agar containing the same reagents as well as 10 mM arabinose at 42°C. CFU were calculated to determine the transformation efficiency.

### Limited Trypsin Digestion Assay

Trypsin cleavage of DnaA was performed as described (Mizushima et al., 1998). DnaA protein was pre-incubated with 2 mM ATP or ADP at 0°C for 15 min and further incubated with 160 ng of trypsin in buffer [50 mM Tricine-KOH (pH 8.25), 0.5 mM magnesium acetate, 0.3 mM EDTA, 7 mM dithiothreitol, 20% (v/v) glycerol, and 0.007% Triton X-100] at 30°C for 30 min. The reaction was terminated by addition of SDS-sample buffer and samples were determined by Western blot analysis.

### Electrophoretic Mobility Shift Assay (EMSA)

Experiments using a minimal *oriC*-containing fragment were performed essentially as previously described (Kawakami et al., 2005). The FAM labeled 469 bp *oriC* was prepared by PCR with the primers 5′ FAM-*oriC* F and 5′ FAM-*oriC* R. The indicated amounts of DnaA protein were incubated for 5 min at 20°C in buffer containing 20 mM HEPES-KOH, pH 8.0, 5 mM magnesium acetate, 1 mM EDTA, 4 mM dithiothreitol, 0.2% Triton X-100, 5% (v/v) glycerol, 0.5 mg/ml BSA, the *oriC* fragment (0.13 pmol) and 2 mM ATP. Reaction products were analyzed by 5% PAGE in cold 0.5xTBE buffer (44.5 mM Tris, 44.5 mM boric acid, 1 mM EDTA) and detected by FUJIFILM FLA7000. Experiments using *dnaA* promoter amplified by 5′ FAM-P*dnaA* F and 5′ FAM-P*dnaA* R were performed in the same way.

Experiments using DARS1 were performed according to published methods (Fujimitsu et al., 2009). DARS1 was amplified using 5′ FAM-DARS1 F and 5′ FAM-DARS1 R, ADP-DnaA was prepared by incubation of DnaA with 2 μM ADP for 15 min at 0°C. ADP-DnaA was incubated for 5 min at 30°C in 12.5 μl of buffer [20 mM HEPES-KOH at pH 7.6, 10 mM magnesium acetate, 1 mM EDTA, 8 mM dithiothreitol, 0.1 mg/ml bovine serum albumin, 5% glycerol, 50 mM potassium glutamate, 2 mM ADP, 21 ng poly(dI–dC), 100 nmol of DARS1].

### *In Vitro* Modification Assay

All *in vitro* modification assays were performed as described (Ren et al., 2016; Zhang et al., 2016). For YfiQ modification assay, DnaA was incubated at 37°C for 6 h in the presence or absence of YfiQ as well as Ac-CoA. For CobB modification assay, DnaA protein was incubated at 30°C for 6 h in the presence or absence of CobB as well as NAD^+^. For AcP modification assay, DnaA was incubated at 37°C in the in the presence or absence of 20 mM AcP, samples were collected at indicated time.

### DNase I Footprint Assay

For preparation of fluorescent FAM labeled probes, the promoter region of *oriC* was PCR amplified with Dpx DNA polymerase (TOLO Biotech) from the plasmid pUC18B-T *oriC* using primers of M13F-47(FAM) and M13R-48. The FAM-labeled probes were purified by the Wizard SV Gel and PCR Clean-Up System (Promega) and were quantified with NanoDrop 2000C (Thermo Scientific).

DNase I footprint assays were performed similar to Wang et al. (2012). For each assay, 700 ng probes were incubated with different amounts of WT and K243Ac in a total volume of 40 μl. After incubation for 30 min at 25°C, 10 μl solution containing about 0.015 u DNase I (Promega) and 100 nmol freshly prepared CaCl_2_ was added and further incubated for 1 min at 25°C. The reaction was stopped by adding 140 μl DNase I stop solution (200 mM unbuffered sodium acetate, 30 mM EDTA and 0.15% SDS). Samples were firstly extracted with phenol/chloroform, then precipitated with ethanol and the pellets were dissolved in 30 μl Milli Q water. The preparation of the DNA ladder, electrophoresis and data analysis were the same as described before (Wang et al., 2012), except that the GeneScan-LIZ500 size standard (Applied Biosystems) was used.

### Western Blot Analysis

Briefly, DnaA protein samples were separated by 10% SDS-PAGE, transferred to PVDF membranes. For acetylation Western blot, 50 mM Tris-HCl (pH 7.5) with100 mM NaCl, 10% (V/V) Tween-20 and 1% peptone (Amresco) was used for blocking. 50 mM Tris-HCl (pH 7.5) with 150 mM NaCl, 0.5% (V/V) Tween-20 and 5% non-fat milk was used for anti-DnaA and anti-His tag Western blot. The rabbit anti-sera against DnaA (1:5000) or anti-AcK (1:1000) or anti-DnaA K243AcK (1:1000) and the mouse anti-sera against-His (1:4000) were used as the primary antibodies and incubated with the membranes overnight at 4°C. Goat horseradish peroxidase (HRP)-conjugated anti-rabbit or anti-mouse IgG antibodies (1: 4000) were used as the secondary antibodies and incubated at room temperature for about 1 h. Blots were scanned with G: BOX Chemi system (Syngene) and relative gray value was quantified by Image J.

### Site-directed Mutagenesis of *dnaA*

Base substitutions were introduced into the wild-type *dnaA* allele by corresponding primers using KOD-Plus-Mutagenesis Kit according to the manufacturer’s instructions (Toyobo, SMK-101). All the site-directed mutants were confirmed by DNA sequencing.

### Identification of Acetylated Lysine Residues by Mass Spectrometry

The purified chromosomally encoded DnaA was separated by 10% one-dimensional SDS-PAGE and the bands containing DnaA were excised. The excised bands were destained and dehydrated. For trypsin digestion, proteins were treated with 100 mM DTT at 56°C for 30 min and then treated with 100 mM NH_4_HCO_3_ at room temperature for 15 min. The freeze-dried samples were incubated with 100–200 ng trypsin at 37°C for 20 h. Peptides generated after proteolytic digestion of DnaA were separated by the EASY-nLC HPLC system (Thermo Scientific) and analyzed by Q-Exactive mass spectrometer (Thermo Scientific). Mass spectrometric data were analyzed using the Mascot 2.2 software for database search.

### The Anti-DnaA K243Ac Specific Polyclonal Antibody Preparation

The peptide AC-CQFFANK(Ac)ERS-NH_2_ conjugated to BSA was used as immune peptide to immunize rabbits. During 2 months, rabbits were immunized for six times, and the antiserum was collected, and control peptide AC-CQFFANKERS-NH_2_ was used to remove non-specific antibody. The sensitivity and specificity of antibody were evaluated by ELISA and Western blot.

## Results

### DnaA K243 Is Required for *In Vivo* DNA Replication Initiation

**FIGURE 1:**
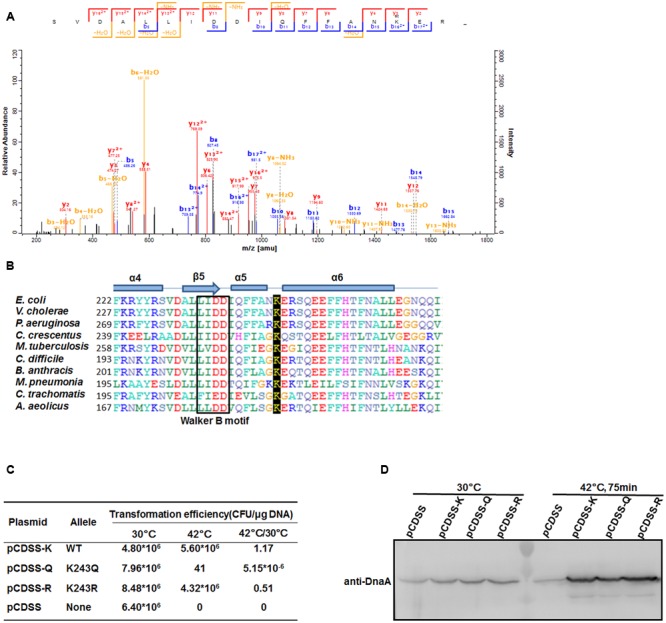
**DnaA K243 is required for *in vivo* DNA replication initiation. (A)** Acetylation of DnaA K243 identified by mass spectrometry. Native DnaA protein was purified and analyzed by liquid chromatography–tandem mass spectrometry after chymotrypsin digestion. Shown is the spectrum covering the region from 200 to 1800 *m*/*z*, which includes the peptide containing the acetylated K243 residue. **(B)** Alignment of DnaA protein sequences. Comparison of *E. coli* DnaA from F222 to I264 with the corresponding DnaA regions from other strains using a Clustal W multiple alignment. Secondary structures are also shown (helices α4–6, sheet β5, and the Walker B motif). *E. coli, Escherichia coli*; *V. cholerae, Vibrio cholerae; P. aeruginosa, Pseudomonas aeruginosa; C. crescentus, Caulobacter crescentus; M. tuberculosis, Mycobacterium tuberculosis; C. difficile, Clostridium difficile; B. anthracis, Bacillus anthracis; M. pneumoniae, Mycoplasma pneumoniae; C. trachomatis; Chlamydia trachomatis; A. aeolicus; Aquifex aeolicus.* **(C)** Plasmid complementation assay. KA413 [*dnaA46* (Ts)] transformed with the indicated plasmids was incubated on LB agar (containing 50 μg/mL thymine and 100 μg/mL spectinomycin) at 30°C, and on LB agar containing the same reagents in addition to 10 mM arabinose at 42°C. Colony-forming units (CFU) were calculated to determine the transformation efficiency and the ratios of CFU at 42°C/30°C are shown. **(D)** Western blot analysis of DnaA. KA413 cells (*dnaA46*) bearing pCDSS (empty vector), pCDSS-K (vector expressing wild-type *dnaA*), pCDSS-Q (vector expressing the K243Q mutant), or pCDSS-R (vector expressing the K243R mutant) were grown at 30°C in LB medium containing thymine (50 μg/mL), spectinomycin (100 μg/mL), and arabinose (10 mM) until the optical density at 600 nm (OD_600_) reached 0.2. A portion (5 ml) of each culture was withdrawn, and the remainder was further incubated for 75 min at 42°C, at which time an additional portion (approximately 1 ml) was withdrawn. Proteins in each sample were subjected to 10% sodium dodecyl sulfate–polyacrylamide gel electrophoresis (SDS-PAGE), and a western blot analysis was performed using an anti-DnaA antibody.

Our previous work showed that DnaA is acetylated and that its acetylation level changes at different growth stages in *E. coli* (Zhang et al., 2016). Here, we purified natively expressed DnaA and identified another acetylated residue, K243 (**Figure 1A**), by mass spectrometry. DnaA is ubiquitous in bacteria (Skarstad and Boye, 1994), and a sequence alignment showed that K243 is highly conserved in eubacteria and archaea (**Figure 1B**). K243 is located between helices α5 and α6 of DnaA, suggesting that its acetylation may affect single-stranded DNA binding by DnaA (Duderstadt et al., 2011). To evaluate the role of K243 acetylation *in vivo*, we performed a plasmid complementation test using *E. coli* strain KA413 (Kawakami et al., 2005), which contains a temperature-sensitive DnaA46 protein that is unstable at 42°C. K243 was mutated either to glutamine (Q), to mimic an acetylated lysine, or arginine (R), to prevent acetylation but retain the positive charge (Ren and Gorovsky, 2001). The plasmids bearing the wild-type or mutant *dnaA* alleles were introduced into *E. coli* strain KA413, colonies of transformants formed with a similar efficiency at 30°C. However, when transformants were incubated at 42°C, the plasmid bearing the *dnaA* K243Q mutation did not support colony formation, while plasmids bearing wild-type *dnaA* or the *dnaA* K243R mutant grew well (**Figure 1C**). To exclude the possibilities of low expression or instability of DnaA K243Q, we determined the DnaA levels in these strains. Western blot analysis showed that the DnaA levels were comparable in the different strains at both temperatures (**Figure 1D**). These results demonstrate that K243 is required for DnaA activity *in vivo*, and suggest that K243 acetylation may be involved in the regulation of DnaA activity.

### Acetylation of K243 Does Not Affect the ATP/ADP Binding Ability of DnaA

**FIGURE 2:**
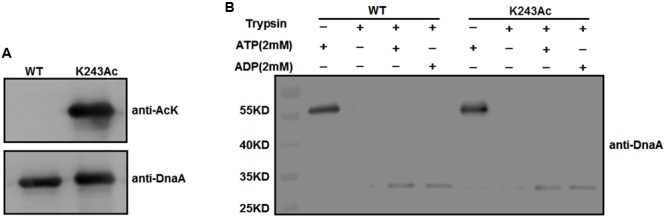
**Purification and ATP/ADP binding activity of DnaA K243Ac. (A)** Immunoblot analysis of DnaA K243Ac. K243Ac was produced by a site-specific acetylation system in the presence of pAcKRS-3 and pCDF-PylT-dnaA (K243TAG). Proteins (purity > 90%) were probed with an anti-acetylated-lysine antibody, and anti-DnaA was used as a loading control. **(B)** ATP/ADP binding activity of DnaA K243Ac. DnaA proteins were preincubated with 2 mM ATP or ADP at 0°C for 15 min and further incubated with 160 ng of trypsin at 30°C for 30 min. The reaction was terminated, and samples were analyzed by Western blot using an anti-DnaA antibody.

To test whether K243 acetylation plays an important role in DnaA activity, we produced a homogeneous, recombinant protein (Supplementary Figure S1) that contains N^𝜀^-acetyllysine at K243 (DnaA K243Ac) using a previously described strategy (Neumann et al., 2008). With high translational fidelity and efficiency, this system has been used widely to decipher the role of acetylation in biological systems (Arbely et al., 2011; Ren et al., 2016; Zhang et al., 2016). Western blot analysis indicated that the acetylation level of K243Ac was much higher than that of the wild-type DnaA (**Figure 2A**).

We have demonstrated that acetylation of the conserved K178 suppresses its binding to ATP/ADP (Zhang et al., 2016). Because K243 is located in the same domain of DnaA as K178, and ATP/ADP binding activity is critical for the function of DnaA, we assessed the binding affinities of DnaA K243Ac using a limited trypsin digestion assay (Mizushima et al., 1998). In the absence of ATP or ADP, DnaA was digested completely by trypsin; however, in the presence of 2 mM ATP or ADP, limited trypsinolysis of the wild-type DnaA and DnaA K243Ac both produced a predominant 30-kDa peptide (**Figure 2B**), which indicates that their binding affinities for ATP and ADP are comparable. These results suggest that K243 is not required for ATP and ADP binding, which is consistent with a previous study demonstrating that a K243A mutation does not affect the ATP/ADP binding affinity of DnaA (Ozaki et al., 2008).

### Acetylation of K243 Inhibits the Formation of the *oriC*/DnaA Complex

**FIGURE 3:**
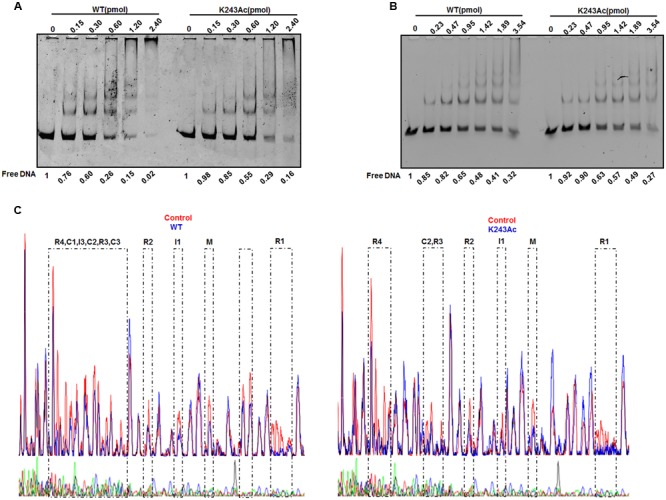
**DNA binding activity of DnaA K243Ac. (A)** *oriC* binding activities assessed by EMSA. Various amounts (0–2.4 pmol) of the wild- type and DnaA K243Ac proteins were incubated with FAM-labeled *oriC* for 5 min at 20°C. Reaction products were analyzed by 5% PAGE and detected using an FLA-7000 image analysis system (Fujifilm, Tokyo, Japan). Free DNA was quantified by Image J. **(B)** The *dnaA* promoter binding activity was determined by EMSA, similar to that used for the *oriC* binding assay, except different DNAs were used. Free DNA was quantified by Image J. **(C)** DNase I footprinting assay. Seven hundred nanograms of FAM-labeled probes were incubated with wild-type DnaA or DnaA K243Ac. After incubation for 30 min at 25°C, approximately 0.015 units of DNase I (Promega, Madison, WI, USA) and 100 nmol of freshly prepared CaCl_2_ were added and further incubated for 1 min at 25°C. The reaction was terminated by adding stop buffer, and the DNA was purified. Electrophoresis and data analysis were performed as described previously (Wang et al., 2012). No protein control and the WT and K243Ac mutant protein profiles were overlaid. The DnaA-binding regions are indicated.

DnaA interacts strongly with DNA. As an initiator, DnaA binds to the replication origin of *E. coli*, resulting in unwinding of an adjacent AT-rich region. In addition to its primary function as the initiator, DnaA also acts as a transcription factor that represses or activates the expression of several genes, depending on the location and arrangement of their DnaA boxes (Messer and Weigel, 1997). We analyzed complexes formed by *oriC* and DnaA using an electrophoretic mobility shift assay (EMSA) (Kawakami et al., 2005) (**Figure 3A**). The results showed that wild-type DnaA formed multimeric complexes with *oriC* DNA in a manner dependent on the amount of DnaA. When DnaA K243Ac was used, we also observed the formation of homomultimers, but more K243Ac was required to form the similar complex pattern, compared with wild-type DnaA. This result indicates that DnaA K243Ac showed a decreased ability in binding *oriC*.

DnaA can auto-regulate its own expression (Braun et al., 1985); thus, we determined its *dnaA* promoter-binding activity in the same way. The results showed that wild-type DnaA and DnaA K243Ac had similar *dnaA* promoter-binding abilities (**Figure 3B**). ADP-DnaA can also bind the DARS to regenerate ATP-DnaA *via* nucleotide exchange (Fujimitsu et al., 2009). Thus, we assessed the binding kinetics of DnaA K243Ac to DARS1 (Supplementary Figure S2), and we found that, similar to the wild-type DnaA, DnaA K243Ac formed complexes with DARS1. These results indicate that K243Ac retains the ability to bind other DNA sequences, except *oriC*.

Because K243Ac showed decreased *oriC* binding activity, we speculate that this mutation may affect the higher-order structure of the *oriC*/DnaA multimer complex. Therefore, a DNase I footprinting assay (Wang et al., 2012) was employed to assess the formation of the ATP-DnaA-specific complex on *oriC*. Similar to the wild-type ATP-DnaA, K243Ac bound to boxes R1, R2, R3, R4, M, I1, and C2, but it failed to recognize the low-affinity boxes I3, C1, and C3 (**Figure 3C**). Additionally, we identified a novel low-affinity site that was bound by the wild-type ATP-DnaA. This site has the sequence 5′–TTAAGATCA–3′, and it is located between boxes R1 and τ1. These results suggest that DnaA K243Ac is defective in forming a complete initiation complex structure, indicating that residue K243 is necessary for the formation of the intact complex.

### The Acetylation Level of K243 Is Regulated by CobB and Acetyl Phosphate (AcP)

**FIGURE 4:**
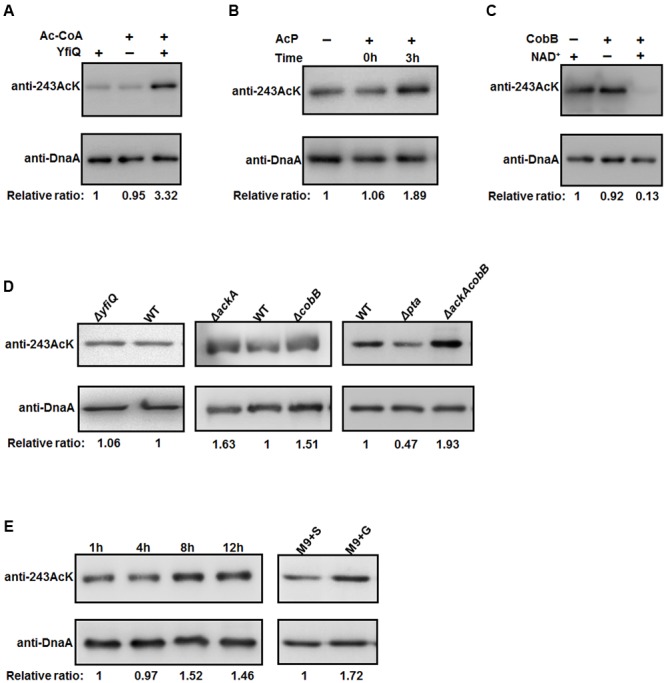
**The acetylation of K243 is regulated by CobB and AcP. (A)** *In vitro* YfiQ modification assay. Wild-type DnaA and DnaA K243Ac were incubated in the presence or absence of YfiQ, as well as the cofactor Ac-CoA. **(B)** *In vitro* AcP modification assay. Wild-type DnaA and DnaA K243Ac were incubated at 37°C in the presence or absence of 20 mM AcP, and samples were collected at the indicated times. **(C)** *In vitro* CobB deacetylation assay. The mutant DnaA K243Ac protein was treated with CobB in the presence or absence of NAD^+^. **(D)** CobB and AcP can regulate the acetylation level of K243 *in vivo*. DnaA protein from the wild-type strain and the indicated deletion strains were cultured in LB medium to the mid-exponential phase and purified. **(E)** The acetylation level of K243 correlates with the intracellular AcP concentration. Cells were grown overnight and then diluted into fresh LB medium supplemented with 0.4% glucose to an OD_600_ of 0.1. Then, the cells were collected at different time points to purify DnaA. Cells grown in M9 minimal medium supplemented with 0.4% glucose (M9+G) or 0.4% succinate (M9+S) to the early stationary phase were also collected to purify DnaA. In **Figure 4**, all the DnaA proteins were resolved on 10% SDS-PAGE and probed with anti-DnaA and anti-DnaA (K243AcK) antibodies. The relative ratios are referred to as the anti-K243Ack: anti-DnaA ratios. Western blots are representative from at least three independent replicates.

To examine the relationship between K243 acetylation and DnaA activity in more detail, it is of interest to identify the factors responsible for regulating the acetylation level of DnaA K243. To do so, we first prepared a K243 site-specific acetylation antibody and confirmed its specificity and sensitivity by Western blot (Supplementary Figure S3). Although *E. coli* contains multiple genes that encode putative acetyltransferases, YfiQ is the only acetyltransferase identified thus far (Starai and Escalante-Semerena, 2004; Liang and Deutscher, 2012), and our previous work demonstrated that YfiQ can acetylate DnaA (Zhang et al., 2016). To determine whether YfiQ can acetylate DnaA K243 directly, the wild-type DnaA was incubated with purified YfiQ and the acetyl group donor acetyl-CoA (Ac-CoA). As shown in **Figure 4A**, the acetylation level of K243 increased significantly, which means that K243 is a substrate of YfiQ *in vitro*. In addition to YfiQ, a non-enzymatic mechanism of acetylation exists, which is dependent on AcP (Weinert et al., 2013; Kuhn et al., 2014). We next examined the involvement of AcP in the acetylation of DnaA K243. Increased acetylation was observed in a time-dependent manner, indicating that AcP can acetylate DnaA K243 *in vitro* (**Figure 4B**). Acetylation can be a reversible and dynamic process that is reversed enzymatically by lysine deacetylase (Starai et al., 2002). As the predominant deacetylase in *E. coli*, CobB plays a major role in the deacetylation of many substrates (Starai et al., 2002; AbouElfetouh et al., 2015). Thus, we purified the K243Ac protein with a high acetylation level, and treated it with CobB in the presence of NAD^+^. The results showed that the acetylation level of K243 decreased significantly after CobB treatment (**Figure 4C**).

We also determined the acetylation level of K243 *in vivo* by purifying native DnaA during the mid-exponential phase from different strains (**Figure 4D**). We found that the acetylation level of K243 in the *yfiQ* mutant was comparable to that in the wild-type strain, which means that YfiQ cannot acetylate K243 *in vivo*. However, in an *ackA* deletion strain, which can accumulate a high level of intracellular AcP (Weinert et al., 2013; Kuhn et al., 2014), the acetylation of DnaA K243 was higher than that of DnaA from the wild-type strain. When the deacetylase CobB was absent, the acetylation of DnaA K243 also increased significantly. These *in vivo* assays were repeated in an *E. coli* strain MG1655 (Supplementary Figure S4), and the results were the same as those in *E. coli* strain BL21. Additionally, the acetylation level of K243 was higher in the *ackA* and *cobB* double deletion mutant than in the single deletion mutants (**Figure 4D**).

To complete the epistasis analysis, we also assessed the acetylation level of K243 in a *pta* mutant. The acetylation level of K243 decreased by approximately 50%, but it was still detectable in the *pta* deletion strain (**Figure 4D**), which cannot produce AcP (Weinert et al., 2013; Kuhn et al., 2014), suggesting the existence of an AcP-independent acetylation mechanism. Taken together, we conclude that the *in vivo* acetylation level of K243 depends on AcP, CobB, and a yet unknown factor, but not YfiQ, although YfiQ could acetylate DnaA on other lysine residues (Zhang et al., 2016). To determine a more precise relationship between the intracellular AcP and acetylation level of K243, we grew the wild-type cells (*E. coli* strain BW25113) in LB broth supplemented with 0.4% glucose, and we harvested the cells at different time points. The signal intensity of K243 acetylation levels increased at 8 and 12 h (**Figure 4E**), which is consistent with a previous report demonstrating that the glucose-induced global acetylation profile increased significantly after entry into stationary phase, at which time AcP begins to accumulate (Schilling et al., 2015). Additionally, cells have a larger acetyl-CoA pool if the carbon source is glucose, rather than succinate (Chohnan et al., 1998); thus, we speculate that the AcP level is higher in a glucose-containing medium. As expected, the acetylation level of K243 was higher when cells were grown in M9 minimal medium supplemented with glucose (M9+G), compared with that in a succinate-supplemented medium (M9+S) (**Figure 4E**). These results demonstrate that the acetylation level of K243 correlates with the intracellular AcP concentration.

## Discussion

**FIGURE 5:**
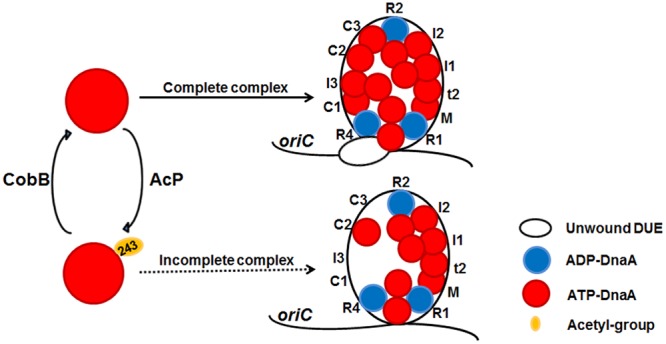
**A model of reversible acetylation of DnaA K243 regulating DNA replication initiation.** AcP and CobB are responsible for regulating the dynamic acetylation level of DnaA K243 *in vivo*. When the acetylation level of DnaA K243 is low, ATP-DnaA can bind to all the DnaA boxes within *oriC*. The formation of a complete initiation complex triggers DUE unwinding. When the acetylation level of DnaA K243 is high, ATP-DnaA fails to bind to several low-affinity DnaA boxes. This incomplete *oriC*-DnaA complex has defect in promoting DUE unwinding.

Like other ATPases associated with diverse cellular activities, bacterial DnaA has a functional conformation that consists of spiral or open-ring-shaped high-order oligomers with a central pore (Ozaki et al., 2008). Certain residues within the pore surface interact directly with *oriC* and play crucial roles in specific DnaA activities. *E. coli* DnaA K243 is located exactly in the central cavity (Ozaki et al., 2008), and we identified its acetylation by mass spectrometry. Our previous study proposed a model in which protein acetylation controls DNA replication initiation by targeting DnaA at the key residue K178 in the Walker A motif. A K178Ac mutant lacks the ability to bind ATP and ADP, and, therefore, it cannot initiate DNA replication (Zhang et al., 2016). In this study, we proposed a working model to display the role of acetylation of DnaA K243 in DNA replication initiation process (**Figure 5**). As a conserved lysine residue located in the same domain of DnaA (**Figure 1A**), the acetylation of K243 did not lower the affinity of DnaA for ATP/ADP (**Figure 2B**), suggesting that K243 acetylation regulates DnaA activity *via* a different mechanism. EMSA assays showed that DnaA K243Ac had a slightly impaired binding to *oriC* (**Figure 3A**), but it bound similarly to other DNAs, such as the *dnaA* promoter and DARS1, compared to wild-type DnaA (**Figure 3B** and Supplementary Figure S2). DNase I footprinting assay revealed that K243Ac cannot recognize several low-affinity boxes, including C1, C2, and I3 (**Figure 3C**). In addition, our DNase I footprinting assay identified a novel site that is bound by the wild-type DnaA, but not by DnaA K243Ac. The novel site is positioned between boxes R1 and τ1, and its sequence is 5′–TTAAGATCA–3′; its role in DNA replication will be investigated in future studies.

Ozaki et al. (2008) reported that a K243A mutant was incapable of binding to ATP-DnaA-specific sites such as the box M and I/τ-sites. This inconsistency is mainly due to the different DnaA mutants used. In our view, K243Ac more accurately represents the true physiological state of the protein, whereas the K243A mutant lacks the lysine side chain completely, which might fully abrogate the function of this residue. Our DNase I footprinting assay did not detect the binding of ATP-DnaA to the box in the 13-mer region, or to the I2 box and τ sites. This difference may be caused by the different DNase I footprinting method that we used, or the DnaA purification methods. ADP-DnaA is unable to form an open complex (Sekimizu et al., 1987; Crooke et al., 1992), although it binds to *oriC* R boxes with the same affinity as ATP-DnaA; therefore, we did not examine the binding activity of ADP-DnaA.

Either the diminished positive charge or the side chain structure of K243Ac inhibits its interaction with the *oriC* region (Wagner and Payne, 2011), especially with low-affinity DnaA binding sites. All these low-affinity DnaA binding sites are required to form replication-efficient pre-replication complexes, and, therefore, they are instrumental for *oriC* unwinding (McGarry et al., 2004; Rozgaja et al., 2011). In addition, the I3 site acts as an important determinant of ATP-DnaA-dependent *oriC* separation, and mutation of a specific position of the I3 site lost its discrimination for ATP-DnaA allowing ADP-DnaA binding (McGarry et al., 2004). Every DnaA box in *oriC* has a function in the initiation process (Langer et al., 1996), therefore, we speculate that the incomplete complex formed by DnaA K243Ac with *oriC* could cause a DNA replication defect.

Lysine acetylation is an abundant post-translational modification in bacteria, and it is involved in a broad range of cellular events such as central metabolism (Wang et al., 2010), transcription (Qin et al., 2016), virulence (Starai and Escalante-Semerena, 2004; Sang et al., 2016), and stress responses (Ma and Wood, 2011). Acetylation is catalyzed by the Gcn5-like acetyltransferase YfiQ (Starai and Escalante-Semerena, 2004) and chemically by AcP (Weinert et al., 2013; Kuhn et al., 2014) in *E. coli*. The NAD^+^ dependent Sir2 homolog CobB (Starai et al., 2002) and the newly found deacetylase YcgC (Tu et al., 2015) can reverse a fraction of the acetylated lysine residues. However, we did not account for YcgC, considering its relatively low expression level (unpublished data). We found that YfiQ can modify DnaA K243 *in vitro* (**Figure 4A**) but not *in vivo* (**Figure 4D**), although it acetylates DnaA at K178. We speculate that purified DnaA may have a different conformation than the native protein *in vivo*, and that YfiQ can access DnaA K243 *in vitro*, but not *in vivo*. Alternatively, the specificity of YfiQ is low and its *in vivo* concentration was not sufficient for DnaA acetylation on K243. Non-enzymatic acetylation with AcP is more global (Weinert et al., 2013; Kuhn et al., 2014; Schilling et al., 2015) compared with YfiQ-catalyzed acetylation, and this small molecule can acetylate DnaA K243 both *in vitro* (**Figure 4B**) and *in vivo* (**Figure 4D**). The deacetylase CobB can effectively remove the acetyl group of DnaA K243 (**Figures 4C,D**), thus, bacteria can dynamically adjust the acetylation level of DnaA K243 in response to environmental stimuli. The detectable acetylation level of K243 in the *pta* mutant strain (**Figure 4D**) demonstrates conclusively that AcP-independent factors are involved in regulating acetylation level of K243. Additionally, we cannot rule out the possibility that another deacetylation pathway exists in addition to CobB. Approximately 70% of the lysine residues of DnaA were identified as substrates for acetylation in *E. coli*, and it is very likely that other acetylated lysine residues have an impact on the activity of DnaA. We believe this reversible, dynamic modification is an efficient way to coordinate the initiation process with environmental changes, which should not be limited to *E. coli.*

## Author Contributions

Conceived and designed the experiments: Y-FY and SL. Performed the experiments: SL and QZ. Analyzed the data: SL and ZX. Contributed reagents/materials/analysis tools: SL, QZ, and ZX. Wrote the paper: SL and Y-FY.

## Conflict of Interest Statement

The authors declare that the research was conducted in the absence of any commercial or financial relationships that could be construed as a potential conflict of interest.
