# Supplementary material for: Acetylation of Lysine 243 Inhibits the oriC Binding Ability of DnaA in Escherichia coli
Source: Front Microbiol. 2017 Apr 20;8:699. doi: 10.3389/fmicb.2017.00699 (PMC5397419; doi:10.3389/fmicb.2017.00699)
Supplement: Supplementary file 1 [file Data_Sheet_1.pdf]

Supplemental information

Table S1 Strains and plasmids used in this study.

**Strains or plasmids**	**Description**	**Sources**
**Strains**		
E. coli DH5α	F- 80*lacZ* M15 (lacZYA–argF)U169 *eoR recA1endA1 hsdR17 phoA supE*44-thi-1 *gyrA96 relA1*	Laboratory stock
E. coli KA413	Ilv thyA tyrA(Am) trpE9829(Am) metE deo supF6(Ts) dnaA46	Gift from T. Katayama
E. coli BL21	F- *ompT gal dcm lon hsdSB* (rB- mB-) λ(DE3 [*lacI lacUV*5-T7 gene 1 *ind1 sam7 nin5*])	Laboratory stock
BL21 derivatives		
his-dnaA	[BL21]*his-dnaA*(cm^R)	Laboratory stock
Δ*cobB-his-dnaA*	[BL21]Δ*cobB-his-dnaA*(cm^R&Kan^R)	Laboratory stock
Δ*yfiQ-his-dnaA*	[BL21]Δ*yfiQ-his-dnaA*(cm^R&Kan^R)	Laboratory stock
Δ*ackA-his-dnaA*	[BL21]Δ*ackA-his-dnaA*(cm^R&Kan^R)	Laboratory stock
Δ*pta-his-dnaA*	[BL21]Δ*pta-his-dnaA*(cm^R&Kan^R)	Laboratory stock
Δ*ackAcobB-his-dnaA*	[BL21]Δ*ackAcobB-his-dnaA*(cm^R&Kan^R)	Laboratory stock
E. coli BW25113	*LacIq rrnBT14* Δ*lacZWJ16hsdR514*	Laboratory stock
	Δ*(araBAD)AH33* Δ*(rhaBAD)LD78*	Laboratory stock
BW25113 derivatives		

his-dnaA	[BW25113]*his-dnaA*(cm^R)	Laboratory stock
Δ*cobB-his-dnaA*	[BW25113]Δ*cobB-his-dnaA*(cm^R)	Laboratory stock
Δ*yfiQ-his-dnaA*	[BW25113]Δ*yfiQ-his-dnaA*(cm^R&Kan^R)	Laboratory stock
Δ*ackA-his-dnaA*	[BW25113]Δ*ackA-his-dnaA*(cm^R&Kan^R)	Laboratory stock
Δ*pta-his-dnaA*	[BW25113]Δ*pta-his-dnaA*(cm^R&Kan^R)	Laboratory stock
**Plasmids**		
pCDSS	Spe^R , used for plasmid complementation assay	Gift from Peter Schultz
pCDSS-K	pCDSSara harboring wild-type *dnaA*	Laboratory stock
pCDSS-Q	pCDSSara harboring *dnaA*(K243Q)	This work
pCDSS-R	pCDSSara harboring *dnaA*(K243R)	This work
pAcKRS-3	Kan^R, coding for *M. barkeri* pyrrolysine tRNA synthetase	Gift from J Chin
pCDF-PylT	Spe^R, derivated from pCDFduet and harboring the *pylT* gene	Gift from J Chin
pCDF-PylT-*dnaA*	Spe^R, used for protein expression	Laboratory stock
pCDF-PylT-*dnaA* (K243TAG)	Spe^R, used for site-specific acetylated DnaA expression	This work
pBKS-*oriC*	Amp^R, derivated from pBluescript II KS(+) and containing the 469-bp *oriC* fragment	Laboratory stock

Table S2 Primers used in this study.

Primer Name	Primer Sequence
K243Q F	CAGTTTTTTGCTAATCAAGAACGATCTCAGGAA
K243Q R	TTCCTGAGATCGTTCTTGATTAGCAAAAAACTG
K243R F	CAGTTTTTTGCTAATCGAGAACGATCTCAGGAA
K243R R	TTCCTGAGATCGTTCTCGATTAGCAAAAAACTG
K243Ac F	CAGTTTTTTGCTAATTAGGAACGATCTCAGGAA
K243Ac R	TTCCTGAGATCGTTCCTAATTAGCAAAAAACTG
pET22b-*dnaA* F	TCTCCATTAGGTGTCACTTTCGCTTTGG
pET22b-*dnaA* R	TCTCCTCGAGCGATGACAATGTTCTGAT
pBKS-*oriC* F	CCGGAGCTCGCCAATGATGATGACGTCAA
pBKS-*oriC* R	GGGGTACCAATCGGTATTGGTAGTCGTG
pCDF-*dnaA* F	CCGCCATGGGCCATCATCATCATCATCATTCACTTTCGCTT TGGCAGCA
pCDF-*dnaA* R	GGCTCGAGTTACGATGACAATGTTCTGATT
5'FAM-*oriC* F	5'FAM-CCCGGGCCGTGGATTCTACT
5'FAM-*oriC* R	5'FAM-CTCGAGGCAGAACTCAAAGA
5'FAM-P*dnaA* F	TGGTCATTAAATTTTCCAATATG
5'FAM-P*dnaA* R	GGCGGACTCCACTCGAAC
5'FAM-DARS1 F	5’FAM-GGGGATAGGGGCTGGAGACA
5'FAM-DARS1 R	5’FAM-CGTGCAAGCCGCGTATTC

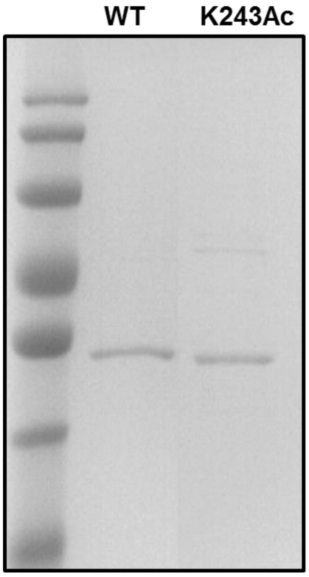

Figure S1. The purified wild-type and DnaA K243Ac proteins. The wild-type and DnaA K243Ac (200 ng) were resolved on 10% SDS-PAGE and stained with Coomassie blue.

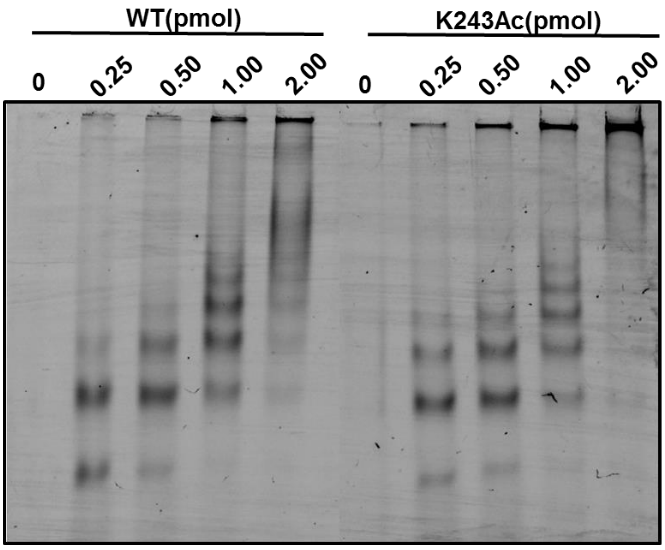

Figure S2. Binding activities of DnaA to DARS1. Various amounts (0-2 pmol) of the wild-type and K243Ac ADP-DnaA proteins were incubated with FAM-labled DARS1 for 5 min at 30℃. Reaction products were analyzed by 5% PAGE and detected by FUJIFILM FLA7000.

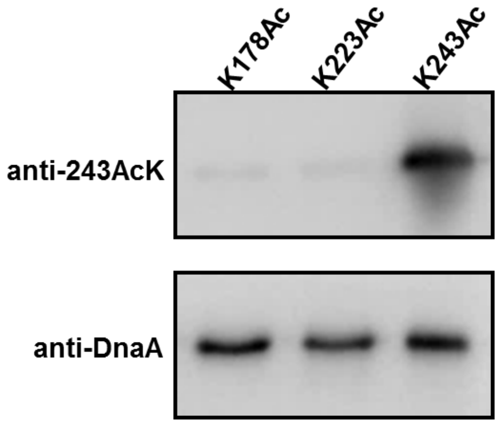

Figure S3. Evaluation of anti-K243Ac antibody by Western blot. Three different site-specific acetylated DnaA were purified, and probed by anti-DnaA antibody and anti-K243Ac antibody, respectively. Western blots were independently repeated at least three times.

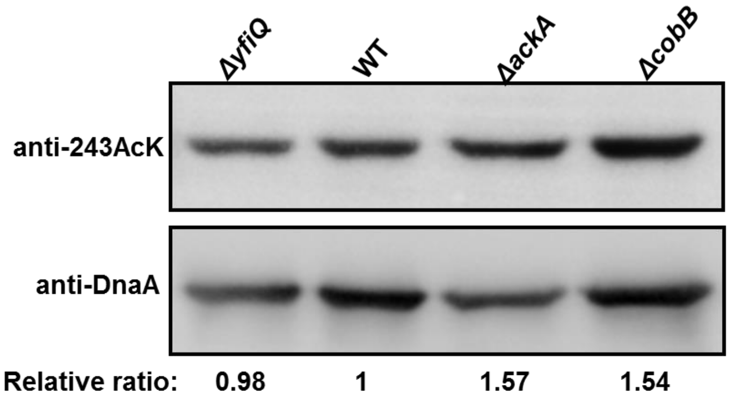

Figure S4. ***In vivo*** **acetylation level of DnaA K243 in different BW25113 strains.** Native DnaA protein was purified from the wild-type *E. coli* strain BW25113 and three mutant strains at the mid-exponential phase, and they were probed by anti-DnaA antibody and anti-K243Ac antibody, respectively. Western blots were independently repeated at least three times.
